# TROP2-directed nanobody-drug conjugate elicited potent antitumor effect in pancreatic cancer

**DOI:** 10.1186/s12951-023-02183-9

**Published:** 2023-11-06

**Authors:** Caili Xu, Min Zhu, Qian Wang, Jiajun Cui, Yuping Huang, Xiting Huang, Jing Huang, Junwei Gai, Guanghui Li, Peng Qiao, Xian Zeng, Dianwen Ju, Yakun Wan, Xuyao Zhang

**Affiliations:** 1 Department of Biological Medicines and Shanghai Engineering Research Center of Immunotherapeutics, School of Pharmacy, Fudan University, Shanghai, 201203 China; 2 Shanghai Novamab Biopharmaceuticals Co., Ltd., Shanghai, 201318 China; 3 Tanwei College, Tsinghua University, Beijing, 100084 China

**Keywords:** TROP2, Nanobody-drug conjugate, Pancreatic cancer, Mechanisms of action, Antitumor effect

## Abstract

**Background:**

Pancreatic cancer is a highly aggressive malignancy with limited treatment options and a poor prognosis. Trophoblast cell surface antigen 2 (TROP2), a cell surface antigen overexpressed in the tumors of more than half of pancreatic cancer patients, has been identified as a potential target for antibody–drug conjugates (ADCs). Almost all reported TROP2-targeted ADCs are of the IgG type and have been poorly studied in pancreatic cancer. Here, we aimed to develop a novel nanobody-drug conjugate (NDC) targeting TROP2 for the treatment of pancreatic cancer.

**Results:**

In this study, we developed a novel TROP2-targeted NDC, HuNb_TROP2-HSA_-MMAE, for the treatment of TROP2-positive pancreatic cancer. HuNb_TROP2-HSA_-MMAE is characterized by the use of nanobodies against TROP2 and human serum albumin (HSA) and has a drug-antibody ratio of 1. HuNb_TROP2-HSA_-MMAE exhibited specific binding to TROP2 and was internalized into tumor cells with high endocytosis efficiency within 5 h, followed by intracellular translocation to lysosomes and release of MMAE to induce cell apoptosis in TROP2-positive pancreatic cancer cells through the caspase-3/9 pathway. In a xenograft model of pancreatic cancer, doses of 0.2 mg/kg and 1 mg/kg HuNb_TROP2-HSA_-MMAE demonstrated significant antitumor effects, and a dose of 5 mg/kg even eradicated the tumor.

**Conclusion:**

HuNb_TROP2-HSA_-MMAE has desirable affinity, internalization efficiency and antitumor activity. It holds significant promise as a potential therapeutic option for the treatment of TROP2-positive pancreatic cancer.

**Graphical Abstract:**

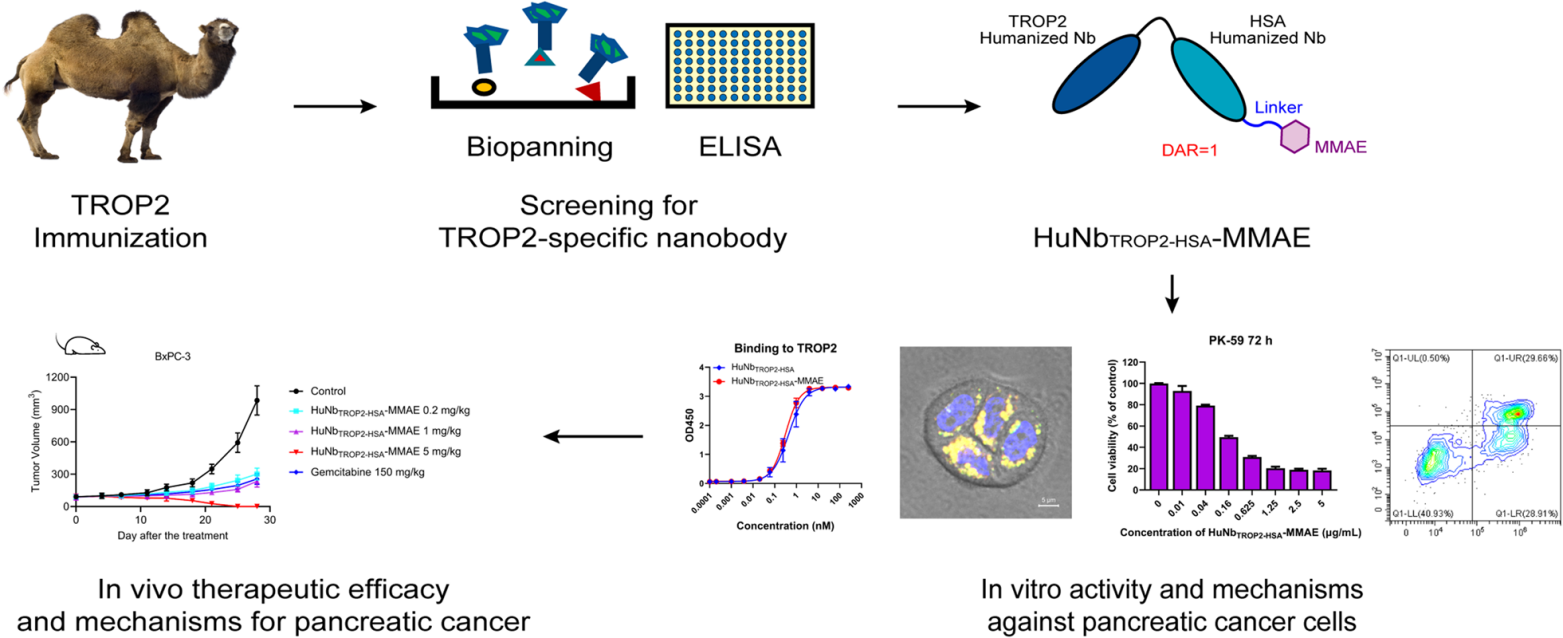

**Supplementary Information:**

The online version contains supplementary material available at 10.1186/s12951-023-02183-9.

## Background

Pancreatic cancer is characterized by its high prevalence, rapid progression and low survival rates. The early symptoms of pancreatic cancer are insidious, often leading to late-stage diagnoses that are beyond the scope of surgical intervention on most occasions. Moreover, the marked aggressiveness and propensity for metastasis pose significant challenges in treatment, resulting in a mere 12% 5-year survival rate among pancreatic cancer patients [1]. Therefore, there is an urgent need for new effective therapeutic targets, strategies and agents.

Trophoblast cell surface antigen 2 (TROP2), also known as tumor-associated calcium signal transducer 2 (TACSTD2), is a transmembrane glycoprotein produced by the TACSTD2 gene. TROP2 was first identified in placental trophoblastic tissue, where it is expressed by cells that possess the ability to invade the uterine wall during placental implantation [2]. In a similar manner, TROP2 may empower cancer cells with a heightened capacity for growth and invasion [3–6]. TROP2 is comprised of an ectodomain, a single transmembrane region and an intracellular tail [7, 8]. Among them, the thyroglobulin type-1 domain in the extracellular region is able to hinder the activity of cysteine proteases and potentially contributes to the metastasis of tumor cells [9]. The intracellular region of TROP2 has a serine residue at position 303 (S303) and a phosphatidyl-inositol 4,5-biphosphate (PIP2) binding site. In the presence of protein kinase C (PKC), S303 is phosphorylated, which promotes the hydrolysis of PIP2 to inositol triphosphate (IP3) and diacylglycerol (DAG). IP3 mediates the release of Ca^2+^ stored in the endoplasmic reticulum, leading to the activation of the mitogen-activated protein kinase (MAPK) pathway, which promotes cell cycle progression [10]. Furthermore, TROP2 has been reported to promote tumor cell growth, proliferation and migration and to inhibit cell adhesion and apoptosis through complex signaling networks [9, 10].

High TROP2 expression was detected in 109 of 197 (55%) pancreatic cancer tumor samples, and TROP2 overexpression was correlated with unfavorable prognosis, as evidenced by decreased overall survival, worse histologic grading and increased incidence of lymph node metastases [5]. Previous research showed that TROP2 expression promoted tumorigenesis in murine subcutaneous and orthotopic pancreatic tumor models and also resulted in more liver metastases [4]. These findings provide theoretical support for the development of pancreatic cancer drugs targeting TROP2 including antibody–drug conjugates (ADCs). Extensive studies have demonstrated the superior antitumor ability of TROP2-targeted ADCs in the treatment of cancers such as breast cancer, lung cancer, and urothelial cancer, and sacituzumab govitecan has received U.S. Food and Drug Administration approval for the treatment of breast cancer and urothelial cancer [8, 9, 11, 12]. However, few systematic studies on TROP2-targeted ADC development for pancreatic cancer have been reported. In addition, the antibody portion of currently reported TROP2-directed ADCs, such as sacituzumab govitecan (IMMU-132), DS-1062, SKB264, ESG-401, DAC-002 and PF-06664178 are all IgG-type monoclonal antibodies, whereas nanobodies have a number of their distinctive advantages that may lead to superior performance of TROP2-directed ADC [9, 12–14].

In this study, we developed a novel TROP2-targeted NDC, HuNb_TROP2-HSA_-MMAE, and evaluated its antitumor activity against human pancreatic cancer. The TROP2-specific antibody used in this NDC is a nanobody screened out based on its high affinity and specificity for TROP2. The humanized TROP2 nanobody was connected to a nanobody against human serum albumin (HSA) and conjugated to monomethyl auristatin E (MMAE) via a lysosomally cleavable linker. To assess the in vitro activity of HuNb_TROP2-HSA_-MMAE, we evaluated its binding activity, observed the internalization, performed cell viability assays and assessed cell apoptosis. Furthermore, we investigated the in vivo efficacy and histological changes in a mouse xenograft model. Our study developed a TROP2-targeted NDC with potent cytotoxicity against TROP2-positive pancreatic cancer, offering new possibilities for the development of more effective and personalized treatments for pancreatic cancer.

## Results

### Screening of anti-TROP2 nanobody

**Fig. 1:**
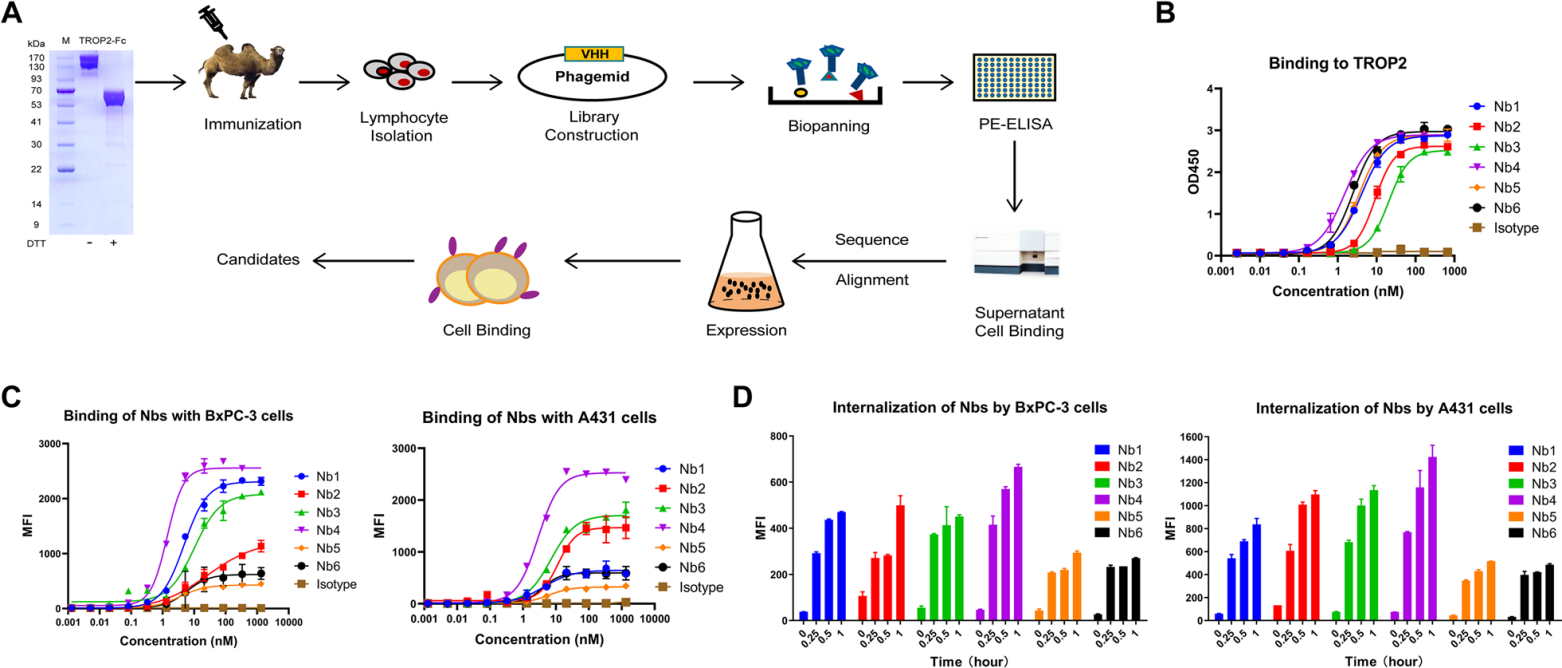
Screening of anti-TROP2 nanobody. **A** Schematic illustration of the screening of TROP2-targeted nanobodies. **B** The affinity of TROP2 nanobody candidates for TROP2 protein was determined by ELISA. **C** Binding activity of TROP2 nanobody candidates to TROP2-expressing cells was assessed by flow cytometry. **D** Cells were incubated with nanobody candidates and the fluorescently labeled secondary antibodies, and the internalization of nanobodies was determined by measuring intracellular fluorescence intensity through flow cytometry

TROP2 antigen was prepared to immunize alpacas to construct TROP2 nanobody phage library, and anti-TROP2 nanobody candidate molecules were obtained via multiplex screening (Fig. 1A). Subsequently, the TROP2 protein was immobilized onto an enzyme-linked immunosorbent assay (ELISA) plate, and the affinity between prokaryotically expressed candidate antibodies and the TROP2 antigen was determined through ELISA experiments. The results showed that Nb1, Nb4, Nb5 and Nb6 exhibited better affinity (Fig. 1B). The binding capability of nanobodies to cell surface antigens was assessed on cells expressing endogenous TROP2 including the human pancreatic cancer cell line BxPC-3 and the human epidermoid carcinoma cell line A431. Consistent results from flow cytometry revealed that Nb4 is the candidate antibody with the highest affinity for cell surface TROP2 (Fig. 1C). Then, the endocytosis of the nanobody candidates was examined. Various concentrations of nanobodies were added to the cell culture medium and incubated at 4 °C for 30 min. After washing, APC anti-HA tag antibodies were added to bind with the nanobodies. After another incubation at 4 °C for 30 min, the cells were transferred to a 37 °C incubator. At different time points, the cells were collected, washed and subjected to fluorescence analysis. As shown in Fig. 1D, Nb4 exhibited the highest internalization in both BxPC-3 and A431 cells. Furthermore, we quantified the precise affinity between Nb4 and hTROP2 using bio-layer interferometry, revealing a KD value of 2.42 nM (Additional file 1: Fig. S1A). The structural prediction and molecular docking based on artificial intelligence displayed the binding site of Nb4 to hTROP2 (Additional file 1: Fig. S1B). Nb4 also showed great performance in stability tests at different temperatures (Additional file 1: Fig. S1C). Based on these data, Nb4, the ideal anti-TROP2 nanobody, was selected for humanization.

### Structure and binding affinity of HuNb_TROP2-HSA_-MMAE

**Fig. 2:**
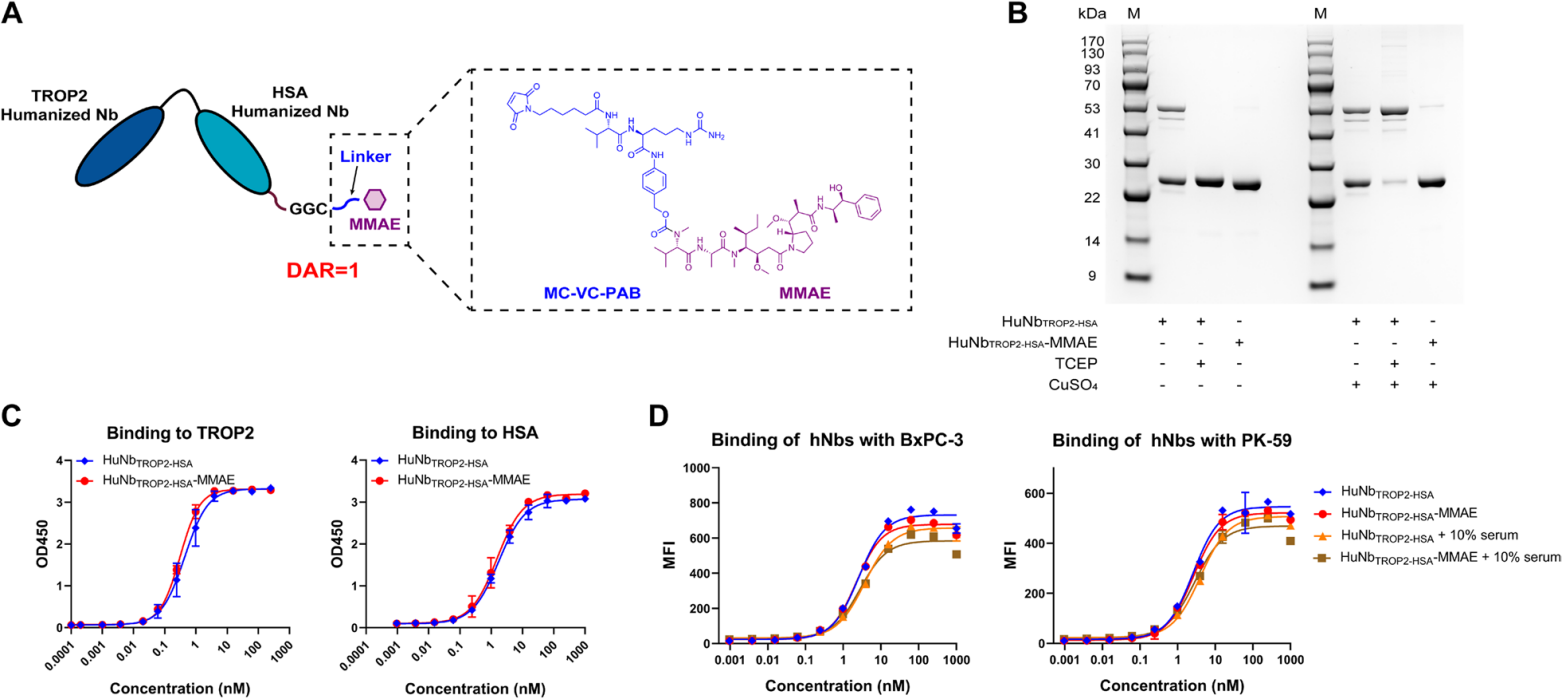
Structure and binding affinity of HuNb_TROP2-HSA_-MMAE. **A** Schematic structure of HuNb_TROP2-HSA_-MMAE. **B** The SDS-PAGE analysis of HuNb_TROP2-HSA_ and HuNb_TROP2-HSA_-MMAE with the addition of the reducing agent TCEP and/or the oxidizing agent CuSO_4_. M represents marker. **C** The affinity of HuNb_TROP2-HSA_ and HuNb_TROP2-HSA_-MMAE for TROP2 proteins was determined by ELISA. **D** Flow cytometry assessed the binding activities of HuNb_TROP2-HSA_ and HuNb_TROP2-HSA_-MMAE to TROP2-positive pancreatic cancer cells in the presence and absence of 10% human serum

The use of nanobodies as the antibody portion of ADCs has numerous advantages. However, nanobodies can be cleared by glomerular filtration, resulting in their short plasma half-life [14–16]. To address this issue, humanized Nb4 was linked to an HSA nanobody to extend the in vivo half-life and the molecule was named HuNb_TROP2-HSA_. Then, HuNb_TROP2-HSA_ and MMAE were conjugated by the MC-VC-PAB linker with a drug-antibody ratio (DAR) of 1 (Fig. 1A and Additional file 2: Fig. S2A). The purity of HuNb_TROP2-HSA_ and HuNb_TROP2-HSA_-MMAE was assessed by SDS-PAGE. Without the addition of CuSO_4_, HuNb_TROP2-HSA_ samples existed as a monomer or dimer, while TCEP-treated HuNb_TROP2-HSA_ was reduced to a monomer and retained the cysteine sulfhydryl group for conjugation. HuNb_TROP2-HSA_-MMAE also appeared as a monomer. After the addition of CuSO_4_, TCEP-treated HuNb_TROP2-HSA_ samples were oxidized to dimers. The majority of HuNb_TROP2-HSA_-MMAE samples remained monomeric, with only a minority existing as dimers that might not have been coupled with MMAE (Fig. 2B). Particle size analysis of HuNb_TROP2-HSA_-MMAE showed a homogeneous particle size distribution with an average hydrodynamic diameter of 3.180 nm (Additional file 2: Fig. S2B).

Then, similar to the previous methods, the affinity of HuNb_TROP2-HSA_-MMAE with TROP2 and HSA was examined by ELISA. The results showed that there was no decrease in the affinity of HuNb_TROP2-HSA_-MMAE compared to that of HuNb_TROP2-HSA_. The EC50 values of HuNb_TROP2-HSA_-MMAE binding to the TROP2 protein and HSA protein were 0.33 nM and 1.57 nM, respectively (Fig. 2C). In regard to its affinity for hTROP2, HuNb_TROP2-HSA_-MMAE displayed a KD value comparable to that of Nb4 (Additional file 2: Fig. S2C). In the assay of binding activity with the human pancreatic cancer cell lines BxPC-3 and PK-59, coupling MMAE did not affect the affinity of humanized nanobodies for cell surface antigens.

Furthermore, HuNb_TROP2-HSA_ and HuNb_TROP2-HSA_-MMAE maintained high affinity in the presence of human serum, which to some extent mimicked the situation encountered by ADC in vivo (Fig. 2D). After 4 days of incubation in human plasma at 37 °C, approximately 85% of HuNb_TROP2-HSA_-MMAE remained intact without premature drug release, ensuring their safety in systemic circulation (Additional file 2: Fig. S2D). Hydrolysis of MC-VC-PAB-MMAE and MMAE release in vivo are dependent on cathepsin B within the cellular lysosomes. Cathepsin B has carboxypeptidase activity and selectively recognizes certain amino acid sequences for the cleavage of specific dipeptide linkages. Therefore, we incubated HuNb_TROP2-HSA_-MMAE with 5 μg/mL cathepsin B to assess the drug release efficiency of HuNb_TROP2-HSA_-MMAE. The results showed that cathepsin B didn’t affect the nanobodies alone, but enabled rapid release of MMAE from NDCs, with more than 60% drug release within 3 h (Additional file 2: Fig. S2E, F).

### Endocytosis and in vitro antitumor activity of HuNb_TROP2-HSA_-MMAE

**Fig. 3:**
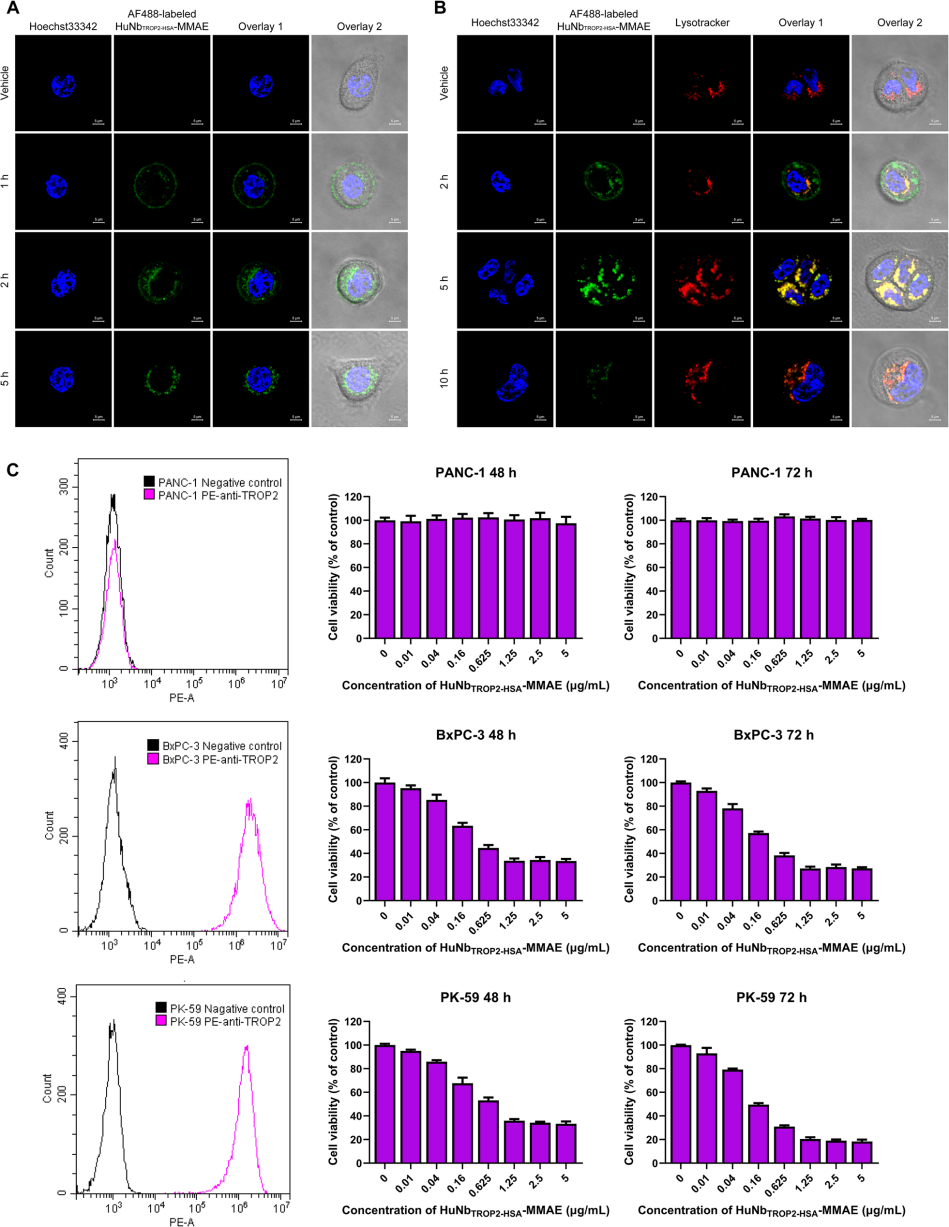
Endocytosis and in vitro antitumor activity of HuNb_TROP2-HSA_-MMAE. **A** The process of HuNb_TROP2-HSA_-MMAE internalization into BxPC-3 cells. Blue: nuclei stained by Hoechst33342; green: HuNb_TROP2-HSA_-MMAE labeled with Alexa Fluor™ 488. **B** HuNb_TROP2-HSA_-MMAE underwent lysosomal degradation after internalization. Blue: nuclei; green: HuNb_TROP2-HSA_-MMAE; red: lysosomes. **C** The expression of TROP2 on the surface of three human pancreatic cell lines was determined by flow cytometry and the cytotoxic effects of HuNb_TROP2-HSA_-MMAE treatment for 2 or 3 days on these cancer cells were evaluated by MTT assay

The internalization of ADC into cells and its subsequent lysosomal degradation leading to the release of cytotoxic drugs are crucial requirements for its therapeutic efficacy. Therefore, we labeled HuNb_TROP2-HSA_-MMAE with AF488 fluorescence and observed endocytosis and lysosomal degradation using confocal microscopy. Figure 3A displayed the entire process of HuNb_TROP2-HSA_-MMAE (marked with green fluorescence) internalization into BxPC-3 cells. At 1 h, HuNb_TROP2-HSA_-MMAE recognized cell-surface TROP2 and was predominantly distributed uniformly on the cell membrane. By 2 h, a significant proportion of HuNb_TROP2-HSA_-MMAE had entered the cells, while some NDCs remained bound to the cell membrane. At 5 h, all NDCs had been internalized into the cells, and no green fluorescence was observed on the cell surface. In the colocalization experiments of NDCs with lysosomes (Fig. 3B), at 2 h, a fraction of internalized NDCs entered the lysosomes, but many NDCs remained in endosomes. At 5 h, lysosomes were highly activated and exhibited a high degree of colocalization with NDCs. After an additional 5 h, the green fluorescence of NDCs was significantly reduced, indicating that they may have undergone lysosomal degradation. These observations demonstrated that HuNb_TROP2-HSA_-MMAE could bind to cell surface antigens, enter cells via antibody-mediated endocytosis, fuse with lysosomes, and undergo lysosomal degradation, which provided assurance of the cellular cytotoxic effect of the payload.

Next, we assessed whether HuNb_TROP2-HSA_-MMAE could exhibit cytotoxicity against target cells. As shown in Fig. 3C, two TROP2-positive human pancreatic cancer cell lines, BxPC-3 and PK-59, as well as one TROP2-negative cell line, PANC-1, were screened out using flow cytometry for subsequent experiments. In the MTT assay, HuNb_TROP2-HSA_-MMAE did not affect the viability of TROP2-negative PANC-1 cells. However, pronounced dose-dependent cytotoxicity was observed in BxPC-3 cells and PK-59 cells. Furthermore, drug treatment for 72 h caused stronger damage to the cells than treatment for 48 h. In addition, cell viability no longer significantly decreased when the NDC concentration exceeded 1.25 μg/mL, indicating that only a low dose of HuNb_TROP2-HSA_-MMAE was required to exert a powerful effect.

### Cell apoptosis induced by HuNb_TROP2-HSA_-MMAE

**Fig. 4:**
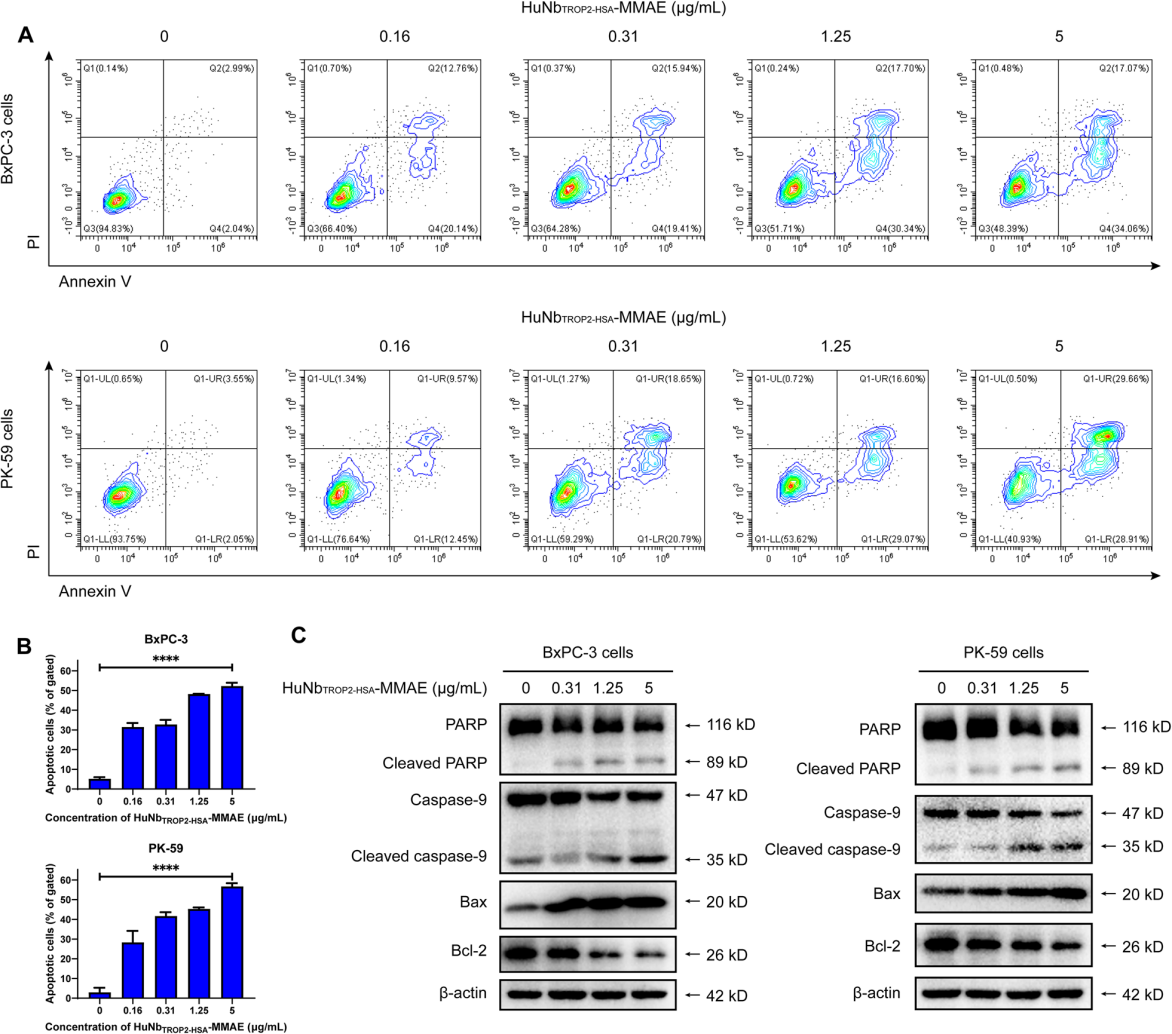
Cell apoptosis induced by HuNb_TROP2-HSA_-MMAE. **A** The effect of HuNb_TROP2-HSA_-MMAE on apoptosis of BxPC-3 and PK-59 cells was detected by Annexin V-FITC/PI staining. Annexin V^+^PI^−^ (Q4) represents early apoptotic cells and Annexin V^+^PI^+^ (Q2) represents late apoptotic cells. **B** A statistical analysis of three replicate experiments of (**A**). The proportion of apoptotic cells is the sum of Q2 and Q4. **C** Western Blot demonstrated the expressions of apoptosis-related proteins in pancreatic cancer cells after 72 h of treatment of HuNb_TROP2-HSA_-MMAE

MMAE causes mitotic inhibition and cell cycle arrest by inhibiting microtubule protein polymerization, which ultimately leads to cell apoptosis. To elucidate the action mechanism of HuNb_TROP2-HSA_-MMAE, the apoptosis of BxPC-3 cells and PK-59 cells was evaluated after incubation with various concentrations of HuNb_TROP2-HSA_-MMAE for 72 h. As shown in Fig. 4A, B, the percentage of Annexin V-positive BxPC-3 cells including early apoptotic cells (Annexin V^+^ PI^−^) and late apoptotic cells (Annexin V^+^ PI^+^) increased in a dose-dependent manner. A similar phenomenon was observed in PK-59 cells. Western Blot was used to further reveal the pathway of HuNb_TROP2-HSA_-MMAE-induced apoptosis. Under the treatment of HuNb_TROP2-HSA_-MMAE, caspase-9 and PARP were activated, thus showing increased cleaved protein bands. Moreover, the pro-apoptotic protein Bax was increased and the anti-apoptosis protein Bcl-2 was decreased. These data suggested that HuNb_TROP2-HSA_-MMAE might initiate apoptosis through activation of caspase-9 and cause cell instability through downstream PARP cleavage.

### In vivo antitumor activity of HuNb_TROP2-HSA_-MMAE

**Fig. 5:**
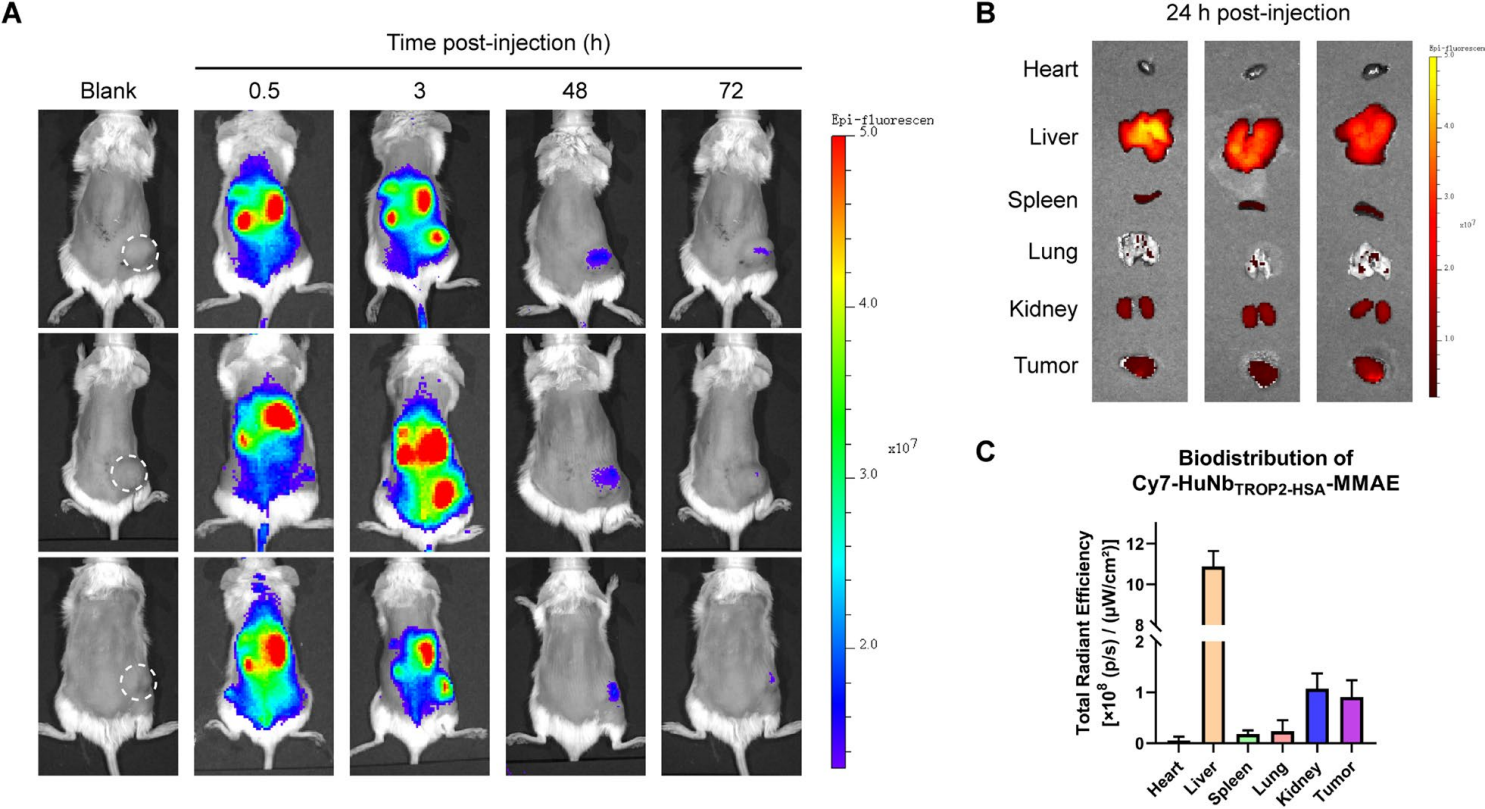
In vivo distribution and metabolism of HuNb_TROP2-HSA_-MMAE. **A** Living imaging after intravenous injection of 200 μg Cy7-HuNb_TROP2-HSA_-MMAE for different times. **B** Fluorescence distribution in major organs at 24 h post-injection. **C** Quantitative analysis of the fluorescence intensity in (**B**)

After confirming the remarkable in vitro antitumor activity of HuNb_TROP2-HSA_-MMAE, we aimed to investigate its role in a mouse model of pancreatic cancer. Firstly, the in vivo distribution and metabolism were examined in a BxPC-3 subcutaneous xenograft tumor model in immunodeficient mice. HuNb_TROP2-HSA_-MMAE was fluorescently labeled by means of conjugation with Cy7 dye. Following intravenous injection of the fluorescently labeled NDC, HuNb_TROP2-HSA_-MMAE rapidly distributed to metabolic organs, such as the kidneys. Three hours post-injection, evident drug infiltration in the tumor was observed (Fig. 5A). At the 24-h time point, a portion of the mice were dissected to observe and quantify the fluorescence intensity of each major organ. As shown in Fig. 5B, C, there was a high distribution of drug in the liver, kidneys and tumor at this time. However, the drug in the metabolic organs was gradually cleared over time, ultimately showing significant enrichment and retention exclusively in the tumor site (Fig. 5A).

**Fig. 6:**
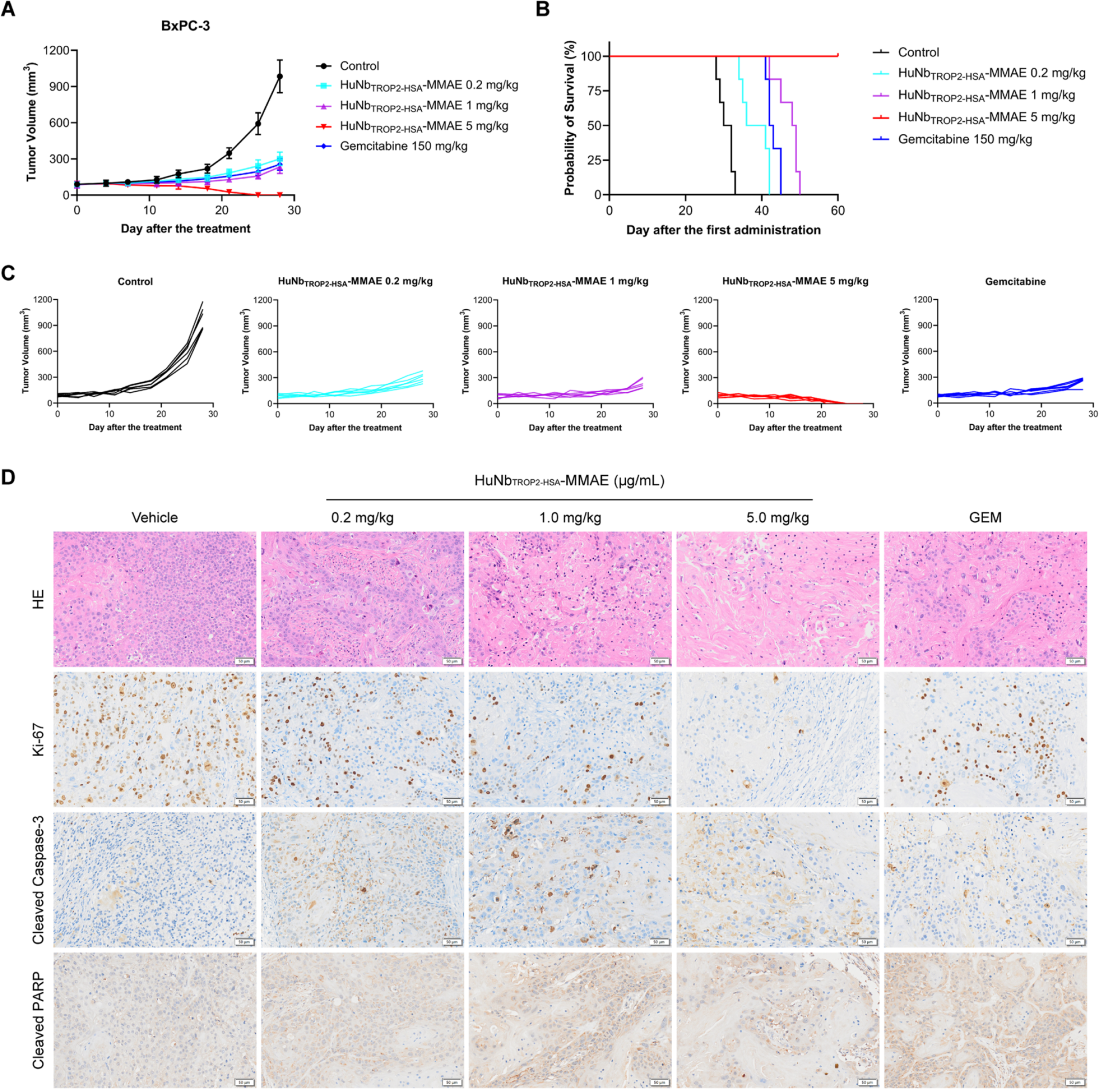
In vivo antitumor activity of HuNb_TROP2-HSA_-MMAE. **A** Tumor volume during 4 weeks of treatment with different doses of HuNb_TROP2-HSA_-MMAE. **B** Survival curves of mice receiving various treatments. **C** Each line represents the tumor volume for an individual mouse. **D** H&E staining and IHC for Ki-67, cleaved caspase-3 and cleaved PARP of tumor tissues

To evaluate the in vivo antitumor activity of HuNb_TROP2-HSA_-MMAE, mice bearing BxPC-3 subcutaneous xenograft tumors were administered different doses (0.2 mg/kg, 1 mg/kg, and 5 mg/kg) of HuNb_TROP2-HSA_-MMAE or the positive control drug gemcitabine twice a week, and tumor volumes were measured over time. As shown in Fig. 6A, C, after 2 weeks of treatment, a gradual difference began to emerge between the ADC-treated groups and the control group. By the end of the third week, one mouse in the 5 mg/kg HuNb_TROP2-HSA_-MMAE group exhibited complete tumor eradication. On day 28 after the first treatment, the average tumor inhibition rates in the low, medium, and high dose HuNb_TROP2-HSA_-MMAE groups were 69.34%, 76.22%, and 100%, respectively, compared to the control group. HuNb_TROP2-HSA_-MMAE at a dose of 0.2 mg/kg already showed significant tumor growth inhibition, while HuNb_TROP2-HSA_-MMAE at 5 mg/kg exhibited even stronger antitumor efficacy, resulting in complete clearance of pre-existing tumors. After the termination of the treatment, survival time was continuously monitored until the mice appeared to die or were sacrificed when the tumor volume reached 1500 mm^3^. The results showed that mice treated with HuNb_TROP2-HSA_-MMAE survived significantly longer, especially in the high-dose group where all mice survived healthily for more than 60 days (Fig. 6B).

To further investigate the effects of HuNb_TROP2-HSA_-MMAE treatment on pancreatic tumors, we collected the tumor tissues from various groups of mice after 2 weeks of treatment and observed the histological changes by H&E staining and immunohistochemistry. In the NDC-treatment groups, especially the high-dose group, a notable reduction in tumor cell population was observed. The nucleoli displayed a smaller size, nuclear division was diminished, and some cells showed nuclear consolidation, nucleolysis, and loss of structure (Fig. 6D). In addition, treatment with HuNb_TROP2-HSA_-MMAE inhibited tumor cell proliferation in a dose-dependent manner, as evidenced by a gradual decrease in the proportion of Ki-67-positive cells (Fig. 6D). We also assessed cell apoptosis by immunostaining for cleaved caspase-3 and cleaved PARP. Cleavage of caspase-3 and PARP is one of the features of cell apoptosis. During the process of apoptosis, caspase-3, once activated through hetero-activation and cleavage, degrades PARP, leading to the loss of its DNA repair function [17]. Our results showed that treatment with HuNb_TROP2-HSA_-MMAE increased cleaved caspase-3 and cleaved caspase-9, suggesting that it induced apoptosis through relevant pathways (Fig. 6D).

## Discussion

It has been documented that TROP2 is highly expressed in a wide range of solid tumors, including breast, lung, ovarian, gastric, colorectal, and pancreatic cancers, but is barely expressed in normal tissues [9, 11, 12, 18]. Higher TROP2 expression is often associated with poorer prognosis, increased tumor aggressiveness and invasion, and resistance to standard therapies [9, 11, 19–21]. Therefore, TROP2 represents an attractive target for the development of novel ADCs. ADCs are formed by coupling cytotoxic drugs to antibodies using a chemical linker. Upon specific binding to tumor antigens and entry into cells via endocytosis, ADCs undergo lysosomal degradation and release cytotoxic drugs that can damage DNA or microtubules, inhibit cell division and cause tumor cell death [22]. Several ADCs targeting TROP2 have been developed in recent years such as IMMU-132, DS-1062, SKB264 and ESG-401 [9, 11, 23–28]. Among these, IMMU-132 has been approved by the U.S. Food and Drug Administration for the treatment of metastatic triple-negative breast cancer (TNBC), metastatic urothelial cancer, and HR^+^/HER2^−^ metastatic breast cancer [24]. Several other representative anti-TROP2 ADCs are in clinical trials for indications involving TNBC, non-small cell lung cancer, ovarian cancer, bladder cancer, and gastric cancer [12, 18, 29]. However, there are few studies on TROP2-directed ADCs for the treatment of pancreatic cancer [18]. Our study assessed the antitumor efficacy of a novel TROP2-targeted NDC, HuNb_TROP2-HSA_-MMAE. The results of our preclinical investigation provide compelling evidence supporting the potential clinical application of TROP2 NDCs in the treatment of TROP2-positive pancreatic cancer.

In the design of ADCs targeting TROP2, factors such as antibody selection, linker chemistries, payload potency, and drug-to-antibody ratio need to be carefully considered to achieve optimal therapeutic outcomes that balance the efficacy and toxicity. In terms of antibodies, currently reported TROP2-directed ADCs, such as IMMU-132, DS-1062, SKB264, ESG-401, DAC-002, FDA018 and PF-06664178 are all IgG-type monoclonal antibodies [9, 12]. Nanobiotechnology has taken on an increasingly pivotal role in tumor diagnosis and treatment, such as nanorobots, nanoprodrugs, and smart transformable nanoparticles [30–36]. In our study, we innovatively developed a nanobody-based TROP2 ADC. Using nanobodies as the antibody portion of ADCs has many advantages, including excellent physical and chemical stability, great aqueous solubility, high tissue penetration, great endocytosis efficiency, and low immunogenicity [14]. Considering the challenge of rapid clearance of the nanobody in the blood, the TROP2 nanobody was coupled with an HSA nanobody to prolong the plasma half-life. This strategy has been successfully applied in Ozoralizumab, the world’s first bispecific nanobody to be approved for marketing [37, 38]. In addition, the DAR and linker are also key factors influencing the efficacy and toxicity of ADCs. For instance, IMMU-132 consists of a humanized antibody hRS7IgG1κ conjugated to the topoisomerase-I inhibitor SN-38 via a CL2A linker with an average DAR of 7.6 [24]. The CL2A linker is hydrolyzed in response to a decrease in the pH of the tumor microenvironment, resulting in the release of SN-38, which kills surrounding tumor cells through a bystander effect. However, high DAR values and unstable linkers increase the risk of causing treatment-emergent adverse events (TEAEs) such as bone marrow suppression, gastrointestinal toxicity, and intermittent lung disease (ILD) [39–43]. Another high-profile TROP2-targeted ADC, DS-1062, which uses an enzymatic linker coupled to Dxd and has a mean DAR of 4, also showed a high rate of serious TEAEs [27]. The incidence of grade 3 or higher adverse reactions in the 6 mg/kg dose group was 48% in phase I clinical trial of non-small cell lung cancer (NSCLC) treatment, and there were even cases of death in the 8 mg/kg dose group, posing a great challenge to the safety of TROP2 ADC. In our study, HuNb_TROP2-HSA_-MMAE has a lysosomally cleavable linker MC-VC-PAB with a DAR value of 1, which may make it have lower systemic toxicity in vivo. Comparisons with previously reported TROP2-targeted ADCs were summarized in Additional file 3: Table S1. should be discussed and summarized in a Table HuNb_TROP2-HSA_-MMAE exhibited desirable binding activity, endocytosis efficiency, inhibition of cell viability, and induction of apoptosis in TROP2-positive pancreatic cancer cells and also showed potent antitumor ability in vivo, which raises new possibilities for the treatment of pancreatic cancer and other TROP2-positive tumors.

## Conclusions

In this work, we demonstrated that nanobody-based TROP2-directed NDC is an effective strategy for the treatment of TROP-positive pancreatic cancer. We developed a novel TROP2-targeted NDC, named HuNb_TROP2-HSA_-MMAE, which consists of a TROP2 nanobody, an HSA nanobody, and MMAE with a DAR of 1. Our research indicated that HuNb_TROP2-HSA_-MMAE had great affinity and internalization efficiency, induced apoptosis and diminished cell viability in vivo, and it inhibited and even eradicated TROP2-positive pancreatic cancer in vivo.

## Materials and methods

### Cell culture

The human pancreatic cancer cell lines BxPC-3 and PANC-1 were purchased from the Type Culture Collection of the Chinese Academy of Sciences, Shanghai, China. PK-59 cells were provided by Shanghai Xingshen Biotechnology Company. The cells were cultured in RPMI-1640 medium with 10% fetal bovine serum, 100 U/mL penicillin and 100 μg/mL streptomycin at 37 °C in a humidified atmosphere containing 5% CO_2_.

### Preparation and screening of TROP2 nanobody candidates

Synthetic TROP2 extracellular domain gene fragments (Sangon Biotech) were synthesized and inserted into the pFUSE vector (Invitrogen). TROP2-Fc was expressed and purified through HEK293F cell expression and then mixed with Freund's adjuvant (Sigma-Aldrich). Two camels were immunized once a week for a total of seven times. Peripheral blood mononuclear cells (PBMCs) were collected, and mRNA was extracted. Subsequently, a phage display library was constructed following the previous protocol. The library capacity and the ratio of correct insertions were calculated. Human TROP2-specific nanobodies were screened through phage display biopanning and periplasmic extract ELISA (PE-ELISA). The positive clone supernatants were further screened by A431 cells, and high-binding activity clones were subjected to sequencing. After removing duplicate sequences, the remaining sequences were synthesized and cloned into the pMECS vector. Nanobodies were expressed by E. coli and purified. All procedures were conducted in accordance with the "Guide for the Care and Use of Laboratory Animals" by the National Institutes of Health in the United States.

### Preparation of HuNb_TROP2-HSA_-MMAE

The identified nanobody against Trop2 was fused with a human albumin-specific nanobody, and a free cystine was designed at the C-terminus in order to conjugate the drug. The constructed bivalent was named HuNb_TROP2-HSA_ and expressed by the *Pichia pastoris* system. The purified HuNb_TROP2-HSA_ was incubated with 10 mM TCEP at 4 ℃ overnight. Two molar equivalents of VcMMAE (MCE, HY-15575) were incubated with TCEP-free HuNbTROP2-HSA at room temperature for 2 h. Then, the reaction was terminated with 20 molar equivalents of acetylcysteine (MCE, HY-B0215), and the buffer was changed to PBS.

### SDS-PAGE

The samples used for loading onto the gel consisted of 5–6 μg of four different variants: HuNb_TROP2-HSA_, HuNb_TROP2-HSA_-MMAE and HuNb_TROP2-HSA_ treated with 10 mM TCEP. In the copper sulfate oxidation treatment, the corresponding samples were treated with 2 mM CuSO_4_ for two hours before loading onto the gel. All samples were prepared and mixed with loading buffer for electrophoresis. After that, the gel was stained with Coomassie Brilliant Blue and underwent several washes to visualize the protein bands.

### Binding activity with antigen proteins

For the determination of the binding activity of nanobody candidates to antigens, the TROP2 protein was diluted to 1 μg/mL in PBS to prepare the coating solution. 100 μL of the coating solution was added to each well of 96-well ELISA plates, followed by overnight incubation at 4 °C. The plates were washed four times with PBST (1 × PBS with 0.05% Tween 20), and then 300 μL of 1% bovine serum albumin (BSA) per well was added and incubated at 37 °C for 2 h. After washing the plate four times with PBST, 100 μL per well of gradient-diluted (in 1% BSA) nanobody was added and incubated at 37 °C for 1 h. The plate was washed four times with PBST, and then 100 μL of the anti-His-HRP antibody (GenScript, A00612) was added to each well and incubated at 37 °C for 0.5 h. After washing the plate four times with PBST, 100 μL of TMB chromogen solution (Biopanda Diagnostics, TMB-S-004) was added. The reaction was carried out at room temperature in the dark for 10–15 min. Then, 50 μL per well of 2 M sulfuric acid solution was used to terminate the color development, and the absorbance (OD450) was measured within 30 min using an ELISA reader (BioTek Synergy) to calculate the EC50 value.

The binding activity of HuNb_TROP2-HSA_ and HuNb_TROP2-HSA_-MMAE to antigens was determined in a similar way as described above. TROP2 and HSA proteins were diluted to 1 μg/mL with PBS as coating solutions to wrap the 96-well ELISA plate. After the abovementioned coating, blocking, drug binding and washing steps, 100 μL/well of biotinylated sheep polyclonal antibody was added and incubated at 37 °C for 1 h. The plate was then washed again with PBST 4 times and incubated with 100 μL/well of streptavidin-HRP (Sigma, S2438, 1:5000) for 0.5 h. Finally, the color development reaction was performed using TMB substrate after washing.

### Stability and drug release analysis

Plasma stability and drug release rate of HuNb_TROP2-HSA_-MMAE were also assessed by ELISA. In the plasma stability tests, 1 mg/mL of HuNb_TROP2-HSA_-MMAE was incubated in human plasma at 37 °C for subsequent testing. In the drug release assays, 0.5 mg/mL HuNb_TROP2-HSA_-MMAE was incubated with 5 μg/mL cathepsin B (MCE, HY-P78682) at pH 6.0 for different time. The hTROP2 protein was coated onto 96-well plates. The NDC samples were incubated with human plasma for various times before being added to the ELISA plate. For total nanobody analysis (including MMAE-containing HuNb_TROP2-HSA_-MMAE and naked nanobodies that have released MMAE), biotinylated sheep polyclonal antibody and streptavidin-HRP were used as described above. For the analysis of naked nanobodies that have released MMAE, each well was added with 100 μL of 200 ng/mL HRP conjugated anti-MMAE antibody (ACROBiosystems, MME-PLS104) to detect only MMAE-containing NDCs. The release of MMAE can be understood by comparing the changes in the concentrations of total nanobodies and NDCs.

### Binding activity with cell surface antigens

The binding activity of purified nanobody monomers was tested in TROP2 endogenously expressing cell lines BxPC-3 and A431. The cells trypsinized and resuspended in complete growth medium. Cells were then washed once with PBS, collected, counted and inoculated into 96-well plates at a density of 2 × 10^5^ cells per well. Diluted antibodies of different concentrations were added and incubated at 4 °C for 40 min. Then, the cells were washed twice with PBS and incubated with 100 μL per well of APC anti-HA.11 Epitope Tag (Biolegend, 901524) at 4 °C for 30 min. Following another round of washing with PBS, the supernatant was removed, and the cells were resuspended in 200 μL per well of PBS for flow cytometry.

### Endocytosis activity assay of anti-TROP2 nanobodies

BxPC-3 cells were seeded into 96-well U-bottom plates at a density of 3 × 10^5^ cells per well. Then, 20 μg/mL nanobody was added to the wells, gently mixed, and incubated at 4 °C for 30 min. After washing the cells twice with PBS, 100 μL of APC anti-HA.11 Epitope Tag (Biolegend, 901524) was added to each well and incubated at 4 °C for 30 min. The cells were washed twice, resuspended in medium and placed in a 37 °C, 5% CO_2_ incubator. At 0, 0.25, 0.5, and 1 h, the experimental groups corresponding to the respective time points were removed and placed in a refrigerator at 4 °C. The cells were then centrifuged at 1600 rpm (4 °C). The supernatant was removed, and the cells were treated with stripping buffer for 8 min. After washing once with PBS, the assay was performed by flow cytometry.

### Cellular TROP2 expression assay

The expression levels of TROP2 on PANC-1, BxPC-3, and PK-59 cells were assessed by flow cytometry. When the cells reached approximately 80% confluence, the culture medium was removed, and the cells were rinsed with PBS followed by detachment using 0.25% trypsin solution without EDTA (Servicebio, G4002-100ML). The trypsin activity was neutralized, and the cell suspension was centrifuged. The resulting cell pellet was resuspended in FACS buffer. The cells were divided into separate tubes for each cell line, and PE anti-TROP2 antibody (Biolegend, 363803) or isotype control antibody was added to the respective tubes. After incubation in the dark at 4 °C for 30 min, the cells were washed and resuspended in FACS buffer for flow cytometry analysis.

### Cell viability assay

The MTT assay was utilized to assess cell viability and cytotoxicity induced by the ADC drug. Cells were seeded in 96-well plates and incubated for 24 h to allow for cell attachment at 37 °C in a 5% CO_2_ humidified atmosphere. The growth medium was replaced with fresh medium containing the ADC drug at different concentrations. After ADC treatment for the desired duration, the medium was removed, and 100 μl of 0.5 mg/ml MTT solution (Meilunbio, MB4698) was added to each well. The plates were incubated for 3 h at 37 °C. Then, the solution was aspirated, and 100 μl of dimethyl sulfoxide was added to each well. The plates were gently shaken for 10–15 min at room temperature for complete solubilization. The absorbance was measured at 490 nm using a microplate reader. Cell viability = (absorbance value of experimental group − blank)/(absorbance value of control group − blank) × 100%.

### Confocal microscopy

Internalization of the ADC drugs and colocalization of ADC with lysosomes were observed by confocal fluorescence microscopy. The ADC drug was labeled with AF488 using the Alexa Fluor® 488 Protein Labeling Kit (Thermo Fisher, A10235). BxPC-3 cells were cultured in confocal dishes and incubated with 1 μg/mL labeled ADC for a specific time. Then, lysosomes were stained with 50 nM LysoTracker™ Red DND-99 (Thermo Fisher, L7528) and nuclei were stained with Hoechst 33,342 (Meilunbio, MA0126). The cells were observed by confocal microscopy (Carl Zeiss, Carl Zeiss LSM710).

### Western blot

Western blot analysis was performed to evaluate the expression levels of apoptosis-related proteins in various cell lines. Cells were washed with ice-cold phosphate-buffered saline (PBS) and then lysed in RIPA buffer (Beyotime, P0013D). The lysates were centrifuged at 12,000 rpm for 10 min at 4 ℃. Then, the supernatants were collected, and the total protein concentrations were determined by a BCA protein assay kit (Beyotime, P0012). After that, proteins were denatured by heating at 95 ℃ for 5 min. Equal amounts of protein were loaded onto SDS-PAGE gels and separated by electrophoresis. Proteins were then transferred onto PVDF membranes. The membranes were blocked with 5% BSA and incubated with primary antibody overnight at 4 °C. After washing four times with TBST, the membranes were incubated with secondary antibody for 1–2 h at room temperature. Protein bands were visualized using an ECL (Meilunbio, MA0186) detection system. The following primary antibodies were used in immunoblot analysis: anti-PARP antibody (Cell Signaling Technology, 9532), anti-Caspase-9 antibody (Cell Signaling Technology, 9502), anti-Bax antibody (Proteintech, 50599-2-ig), anti-Bcl2 antibody (Proteintech, 12789-1-ap), and anti-β-actin antibody (Servicebio, GB15003-100).

### Apoptosis analysis

The Annexin V-FITC/PI Apoptosis Detection Kit (Meilunbio, MA0220) was used to evaluate cell apoptosis. Cells were treated under experimental conditions and harvested. The cells were then resuspended in 1 × binding buffer and stained with Annexin V-FITC and PI. After incubation, the cells were analyzed using a flow cytometer (Beckman, CytoFLEX), and appropriate gating strategies were applied to determine the percentage of apoptotic cells. Flow cytometry data were analyzed using CytExpert software to quantify the different cell populations.

### Xenograft models

All experimental procedures involving animals were conducted in strict accordance with the guidelines and protocols approved by the Animal Ethical Committee at the School of Pharmacy, Fudan University. BxPC-3 cells were subcutaneously engrafted into female NCG mice (Shanghai Model Organisms Center Inc., China). When the average tumor volume reached approximately 90 mm^3^, the mice were randomly divided into 5 groups, with 6 mice per group. The mice were intravenously injected with vehicle or the testing item twice a week and tumor dimensions (long and short diameters) were measured. The doses of HuNb_TROP2-HSA_-MMAE were 0.2, 1, and 5 mg/kg. The positive drug group was given 150 mg/kg gemcitabine (MCE, HY-17026). The formula used for calculating tumor volume was 1/2 × (long diameter in mm) × (short diameter in mm) × (short diameter in mm).

### Immunohistochemistry

After 2 weeks of treatment, tumor tissues were collected and fixed in 4% paraformaldehyde solution (Servicebio, G1101) for 24 h and subsequently embedded in paraffin. Then, they were sectioned at a thickness of 4–5 μm using a microtome and mounted on glass slides. Prior to IHC staining, the sections were deparaffinized in xylene and rehydrated through a graded ethanol series (100%, 95%, 70%, and 50%) followed by washing in distilled water. After that, the sections were subjected to heat-induced epitope retrieval in 10 mM sodium citrate buffer (Servicebio, G1219). After cooling to room temperature, endogenous peroxidases were quenched with 3% hydrogen peroxide in PBS for 10 min. The sections were then incubated in 5% BSA for 1 h. Next, the tumor tissue sections were incubated overnight at 4 °C with primary antibodies against Ki-67 (Servicebio, GB121499-100), cleaved PARP (Servicebio, GB111503-100), and cleaved caspase-3 (Servicebio, GB11532-100) at the appropriate dilutions. After washing with PBS, the sections were incubated with HRP-conjugated secondary antibodies for 1 h. Diaminobenzidine (Servicebio, G1212-200T) was used as the chromogen to visualize the antibody-antigen complexes, and sections were counterstained with hematoxylin. Finally, the sections were sealed and photographed through a microscope.

### H&E staining

Tumor tissue slices were obtained as described above. The slices were then deparaffinized and rehydrated through a series of xylene and ethanol baths. Hematoxylin and Eosin (H&E) staining was performed, with the former used to stain nuclei and the latter to stain cytoplasm and other tissue components. After staining, the tissue slices were dehydrated, cleared, and coverslipped using a mounting medium. Histopathological analysis was conducted by examining the slices under a light microscope.

### Bio-layer interferometry

The affinity of Nb4 to recombinant human TROP2 were determined by an fortebio octet system (ForteBio, Menlo Park, CA, USA). Briefly, the streptavidin A biosensors were wetted in PBST buffer for about 10 min. The diluted biotinylated TROP2 protein was coupled to the streptavidin A biosensors. Thereafter, it was incubated with serially diluted Nb4 and then dissociated in PBST buffer. The binding curve and the equilibrium dissociation constant (KD) was analyzed by ForteBio Data Analysis 9.0 software.

### Molecular docking

The interaction of the TROP2 extracellular domain with Nb4 or Trodelvy® HC variable domain was predicted by AlphaFold2.

### Mass spectrometry

The DAR of HuNb_TROP2-HSA_-MMAE was determined using Matrix-Assisted Laser Desorption/Ionization Time-of-Flight Mass Spectrometry. The NDC sample was transferred to a pure aqueous solution by ultrafiltration and then subjected to rapifleX® for mass spectrometry analysis according to the instrument instructions.

### Measurement of hydrodynamic diameter

The particle size of HuNb_TROP2-HSA_-MMAE was measured by Zetasizer Nano ZSP. In brief, 2 mg/mL of HuNb_TROP2-HSA_-MMAE dispersed in PBS solution was added to the cuvette (ZEN0040) and placed in the instrument for three repetitions for particle size analysis.

### Protein fluorescent labeling and imaging

To prepare fluorescently labeled proteins, 1 mg Sulfo-Cy7 (DuoFluor, D10018) was dissolved in 100 μL of dimethyl sulfoxide. Then, 50 μL of 10 mg/mL Sulfo-Cy7 was added to 1 mL of 2 mg/mL HuNb_TROP2-HSA_-MMAE and incubated for 4 h at room temperature with slight oscillation away from light. Next, free Sulfo-Cy7 dye was removed by multiple ultrafiltration. The activity of Cy7-HuNb_TROP2-HSA_-MMAE passed quality control. For each mouse bearing BxPC-3 subcutaneous tumors, 200 μg of Cy7-HuNb_TROP2-HSA_-MMAE was intravenously administered via the tail vein. In vivo imaging of the mice at different time intervals was carried out using the IVIS® Imaging System (PerkinElmer). Following a 24-h post-injection period, three mice are euthanized, and their major organs were dissected for imaging. The data were analyzed by Living Image® 4.4 software.

### Statistical analysis

Data analysis was conducted using GraphPad Prism 9 software. The results were shown as the mean ± SD. Differences between two groups were assessed using Student’s *t* test, and comparisons among multiple groups were performed using one-way ANOVA. *P* values were presented using the following asterisk rating system: *P* < 0.05 *, *P* < 0.01 **, *P* < 0.001 ***, and *P* < 0.0001 ****.

### Supplementary Information

**Additional file 1: Fig. S1**. Characterization of Nb4. (**A**) The affinity of Nb4 for hTROP2 was estimated by bio-layer interferometry. (**B**) Molecular docking simulation of Nb4 to the extracellular region of hTROP2. (**C**) The stability of Nb4 was revealed by detecting the affinity of Nb4 for hTROP2 by ELISA after being placed at different temperatures for 1 week.

**Additional file 2: Fig. S2**. Characterization and stability of HuNbTROP2-HSA-MMAE. (**A**) The DAR of HuNbTROP2-HSA-MMAE was determined by mass spectrometry. (**B**) The hydrodynamic diameter of HuNbTROP2-HSA-MMAE. Each color represents one independent repetition of the experiment. (**C**) The affinity of HuNbTROP2-HSA-MMAE for hTROP2 was estimated by bio-layer interferometry. (**D**) The stability of HuNbTROP2-HSA-MMAE in human plasma was determined by ELISA. (**E**) Changes in total anti-TROP2 nanobodies were detected by ELISA after incubating HuNbTROP2-HSA-MMAE with cathepsin B for different times at 37 °C. (**F**) The rate of MMAE release from HuNbTROP2-HSA-MMAE incubated with cathepsin B was measured by ELISA.

**Additional file 3: Table S1**. Comparison of TROP2-targeted ADCs.

## Data Availability

All data generated or analyzed during this study are included in the article.

## Abbreviations

ADC: Antigen-drug conjugate
BSA: Bovine serum albumin
DAG: Diacylglycerol
DAR: Drug-antibody ratio
HSA: Human serum albumin
H&E: Hematoxylin and eosin
ILD: Intermittent lung disease
IP3: Inositol triphosphate
MAPK: Mitogen-activated protein kinase
MMAE: Monomethyl auristatin E
NDC: Nanobody-drug conjugate
PBMC: Peripheral blood mononuclear cell
PE-ELISA: Periplasmic extract enzyme-linked immunosorbent assay
PI: Propidium iodide
PIP2: Phosphatidyl-inositol 4,5-biphosphate
PKC: Protein kinase C
TACSTD2: Tumor-associated calcium signal transducer 2
TEAE: Treatment-emergent adverse event
TNBC: Triple-negative breast cancer
TROP2: Trophoblast cell surface antigen 2
