# Supplementary figures and images for: TROP2-directed nanobody-drug conjugate elicited potent antitumor effect in pancreatic cancer
Source: J Nanobiotechnology. 2023 Nov 6;21:410. doi: 10.1186/s12951-023-02183-9 (PMC10629078; doi:10.1186/s12951-023-02183-9)

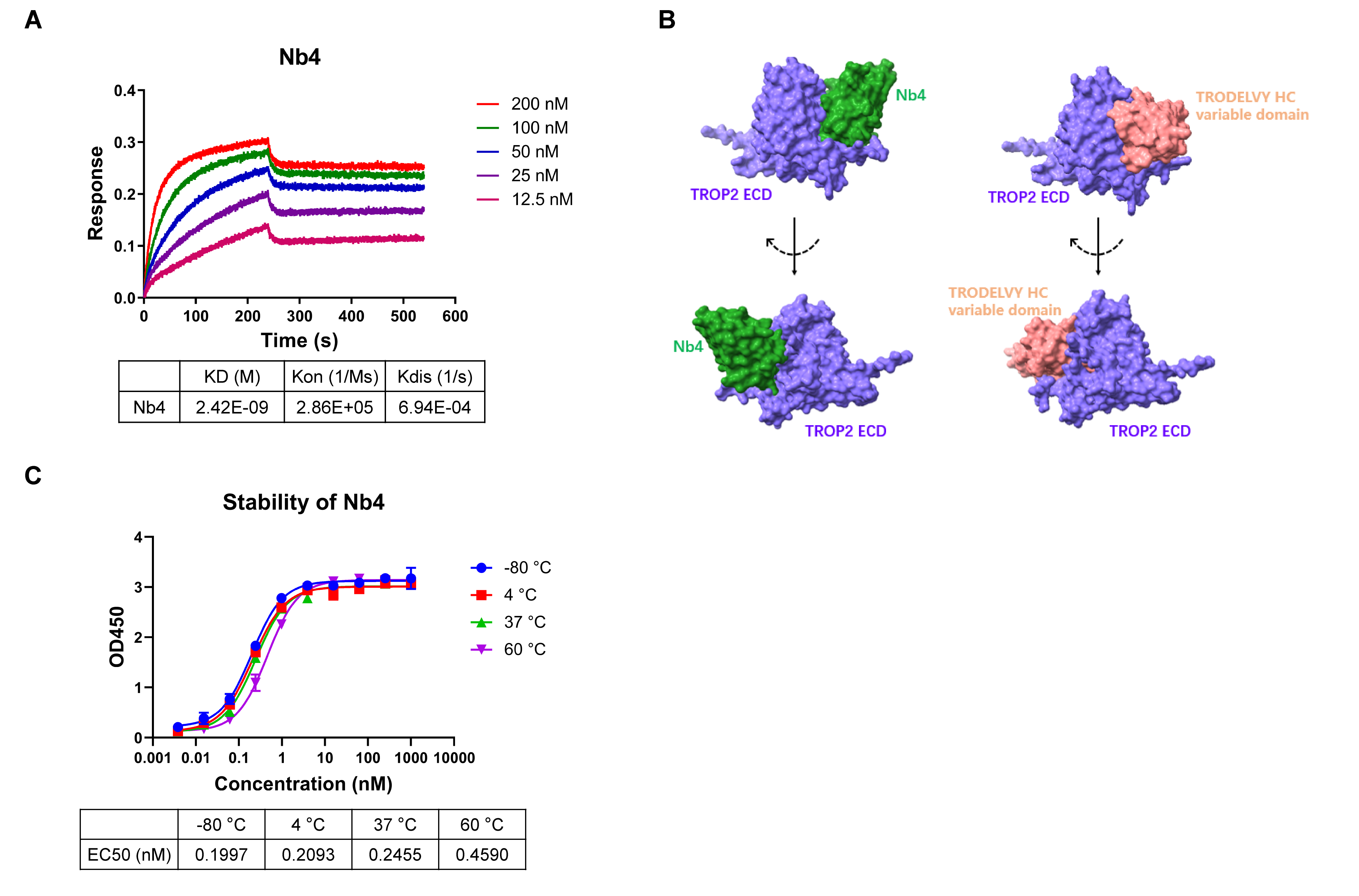

Supplement: Supplementary file 1 — Additional file 1: Fig. S1. Characterization of Nb4. (A) The affinity of Nb4 for hTROP2 was estimated by bio-layer interferometry. (B) Molecular docking simulation of Nb4 to the extracellular region of hTROP2. (C) The stability of Nb4 was revealed by detecting the affinity of Nb4 for hTROP2 by ELISA after being placed at different temperatures for 1 week. [file 12951_2023_2183_MOESM1_ESM.tif]

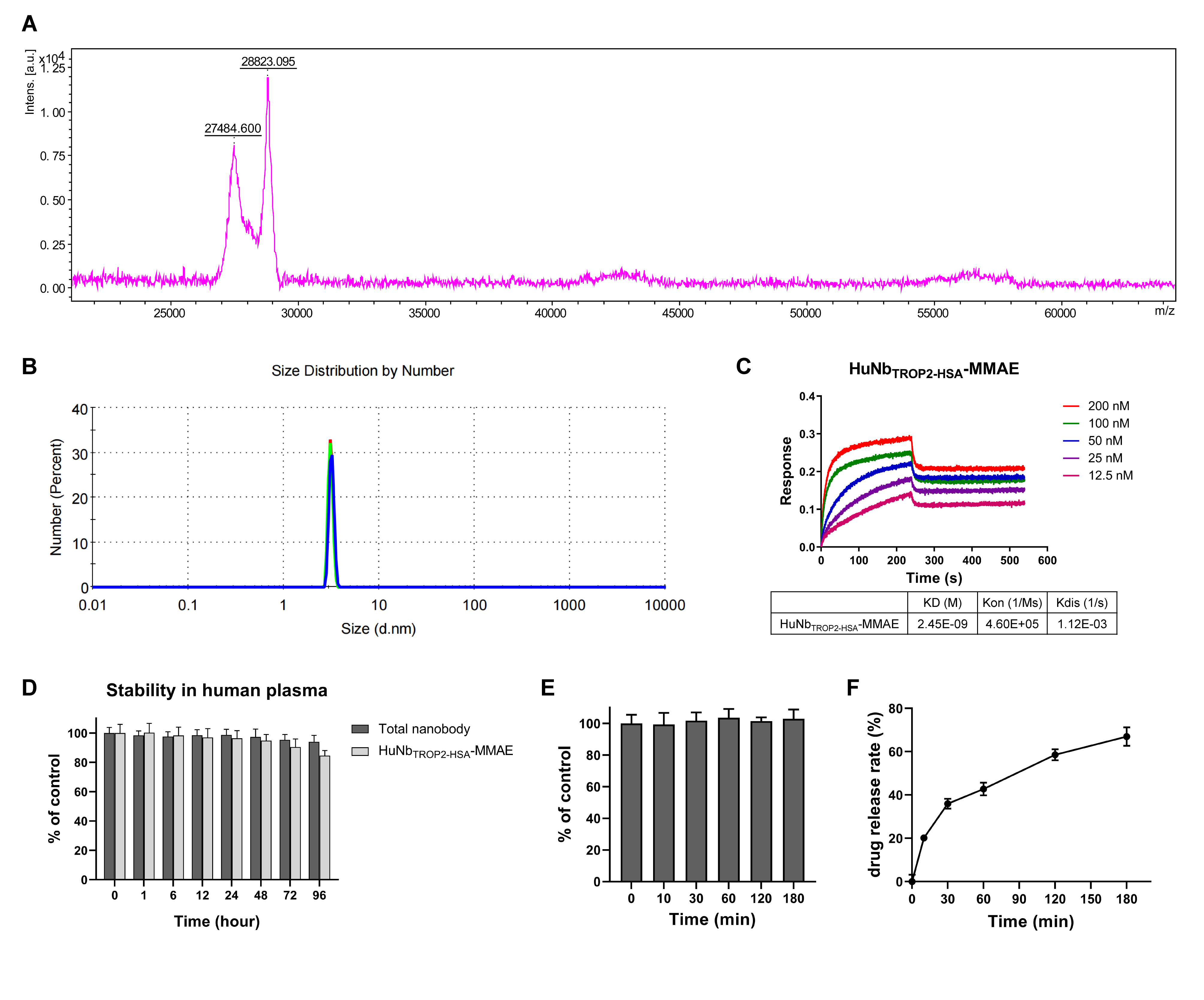

Supplement: Supplementary file 2 — Additional file 2: Fig. S2. Characterization and stability of HuNbTROP2-HSA-MMAE. (A) The DAR of HuNbTROP2-HSA-MMAE was determined by mass spectrometry. (B) The hydrodynamic diameter of HuNbTROP2-HSA-MMAE. Each color represents one independent repetition of the experiment. (C) The affinity of HuNbTROP2-HSA-MMAE for hTROP2 was estimated by bio-layer interferometry. (D) The stability of HuNbTROP2-HSA-MMAE in human plasma was determined by ELISA. (E) Changes in total anti-TROP2 nanobodies were detected by ELISA after incubating HuNbTROP2-HSA-MMAE with cathepsin B for different times at 37 °C. (F) The rate of MMAE release from HuNbTROP2-HSA-MMAE incubated with cathepsin B was measured by ELISA. [file 12951_2023_2183_MOESM2_ESM.tif]
